# What Can Systems Theory of Networks Offer to Biology?

**DOI:** 10.1371/journal.pcbi.1002543

**Published:** 2012-06-28

**Authors:** Indika Rajapakse, Mark Groudine, Mehran Mesbahi

**Affiliations:** 1 Division of Basic Sciences, Fred Hutchinson Cancer Research Center, Seattle, Washington, United States of America; 2 Department of Biostatistics and Biomathematics, Public Health Sciences, Fred Hutchinson Cancer Research Center, Seattle, Washington, United States of America; 3 Department of Radiation Oncology, University of Washington School of Medicine, Seattle, Washington, United States of America; 4 Department of Aeronautics & Astronautics, University of Washington, Seattle, Washington, United States of America; Indiana University, United States of America

Cell function manifests through intricately controlled dynamic processes that both interact amongst each other—in a network—and also respond to external signals, e.g., transcription factors, etc. Recently, controllability of complex networks has received considerable attention for its potential to influence the behavior of dynamically interacting nodes of interest in biological, social, and engineered networks. For example, Liu et al. recently used the notion of structural controllability to provide insights into the percentage of nodes required to control the evolution of the state of the nodes in a network in response to external stimuli [1]–[3]. However, when applying the notion of controllability in biology, we must consider the dynamical nature of biological systems as well as the timing of the external input. Here, we provide a parallel yet distinct approach to the problem of network controllability in the context of cell differentiation and highlight the importance of network controllability and its applications in dynamical biological systems [4].

Cellular differentiation is the process via which the nucleus achieves a new function. After many years of intensive investigation, we are just scratching the surface of how the cell nucleus functions on a global level to produce specialized cell types. The relationship between nuclear form and function will be critical to understanding the dynamics of the nucleus during cell differentiation [5], [6]. We argue that feedback between form and function during differentiation fine-tunes a cell-specific form, leading to the desired function [7]. In this venue, we first define form as a geometric network that reflects the physical configuration of chromosomes in the interphase nucleus and function as the transcriptional network. Adopting this geometric point of view allows us to consider the evolution of a concrete, physically realizable network during differentiation, and assess how essential features of this network evolve over time. The basic question is thus how network geometry with a particular initial configuration evolves toward a specific cell type with its own unique configuration and how essential features of the network evolve with this geometry.

Two constructs form the basic premise in addressing this question. First, cell fate is guided by transcription factors that have broad influence on cell differentiation and reprogramming [8]. An example of this type of influence is the well-studied transcription factor MyoD, which can convert fibroblasts into skeletal muscle cells by activating the skeletal muscle differentiation program [9]. Another example is the recently discovered set of transcription factors that can reprogram cells to pluripotency [10]. Second, we pose that the essential features of the network that evolve to allow for efficient reprogramming fall in the realm of control theory.

One of the basic concepts in control theory is—to no surprise—controllability. Essentially, controllability is a feature of a dynamic system with inputs, allowing its states to be steered towards desirable target states. If we now consider the physical configuration of the chromosomes, the form, as the dynamic state during differentiation, and transcription factors such as MyoD as the external signals, the natural idea is to examine the differentiation dynamics from the point of view of its *controllability*. What would such a point of view offer? It provides a systematic means of quantifying directions that the network can most efficiently be steered towards, given how the transcription factor binds and interacts with various parts of the chromosomal network. This can be accomplished by not only considering whether the network is controllable from the input, but also examining how controllable each direction in the configuration space of the chromosomal network is. And since the network is dynamic—after all, that is what differentiation is all about—a static, parameterized notion of controllability, such as structural controllability, turns out to be unsuitable for assessing network controllability. In the structural controllability approach, the weights on the interactions are of little consequence—as long as they are zero and non-zero—for assessing whether the network is steerable to a particular configuration or not. However, in networks such as the chromosomal network during cellular differentiation, there is intriguing evidence that two nodes interact more if their pairwise physical distance is less. Thus, as the nodes in the chromosomal network come closer, they have a higher interaction with each other, and it is well-conceivable that the network becomes less controllable by an external signal such as a transcription factor.

**Figure 1:**
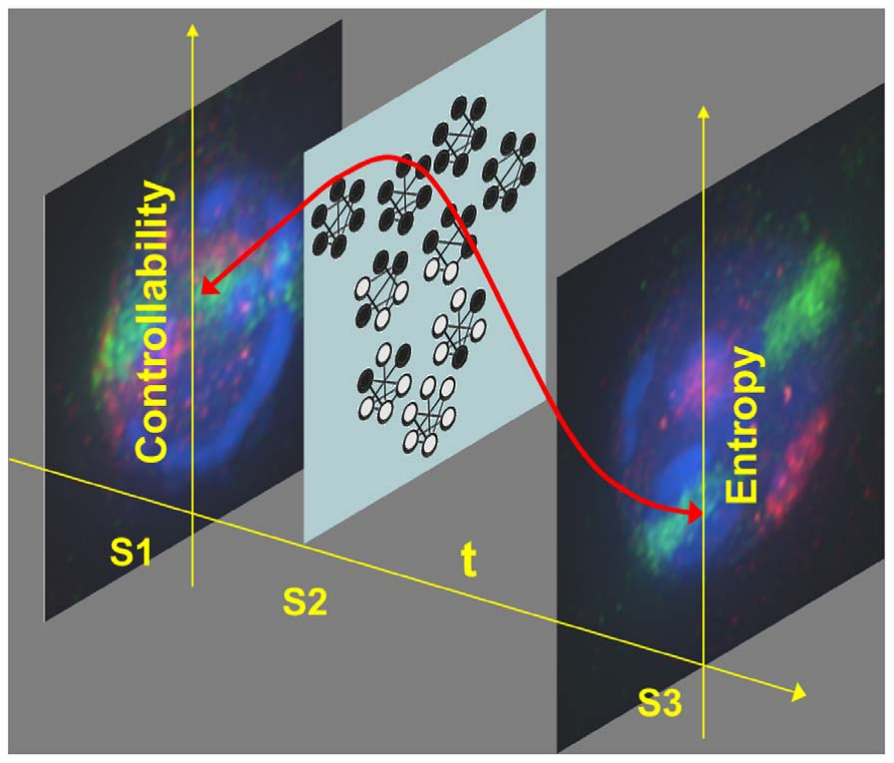
Dynamics of network controllability and entropy. The process of cell differentiation as a dynamical system varies in degree of controllability, or receptivity to external signals at particular stages, where cells pass through an intermediate highly receptive and entropic state. S1 (state 1, stem/progenitor state), S2 (state 2, transition state), S3 (state 3, terminally differentiated state).

We have proposed a framework to examine this notion of controllability via the controllability gramian, a matrix that captures how directions in the nodes' configuration space are attainable with a given external input [11]. The controllability gramian not only reveals the efficiency and time critical nature of steering the network, but also has a statistical significance in terms of its connection with measurement covariances and their determinant, the network entropy. Thus, we can estimate the entropy from the determinant of the covariance matrix, and interpret it as a measure of network controllability. Using this approach, we have proposed a diffusion-driven network with dynamics that is parameterized by the relative distances between the nodes, for which the evolution of the controllability gramian not only matches the experimentally determined covariances in terms of their algebraic properties, but also offers a control theoretic parallel for a highly entropic intermediate network as the cell transitions between two highly ordered states during differentiation (Figure 1).

The dynamical properties of a cell are hardwired in the genome, but both environmental and epigenetic factors influence how this information is accessed and applied [12], [13]. Thus, a cell is naturally receptive to external influence, and this gives us an opportunity by which to manipulate a cell to achieve a desired function or outcome. To best take advantage of this property, we require a deeper understanding of when and where to apply external influences. The application of network controllability theory may be the key to systematic reasoning about which nodes to target to achieve global impact toward a desired outcome, and when to target them in a perturbed system, such as cancer. This may lead to novel strategies for redirecting cancer cells along new trajectories that avoid further pathology. However, the dynamic nature of the network, as well as the efficiency and timing of the control mechanism, should be an integral part of such a network controllability research.
